# Application of Taguchi method and response surface methodology into the removal of malachite green and auramine-O by NaX nanozeolites

**DOI:** 10.1038/s41598-021-95649-5

**Published:** 2021-08-06

**Authors:** Siroos Shojaei, Saeed Shojaei, Shahab S. Band, Amir Abbas Kazemzadeh Farizhandi, Milad Ghoroqi, Amir Mosavi

**Affiliations:** 1 Department of Chemistry, Faculty of Sciences, University of Sistan and Baluchestan, Zahedan, 98135-674 Iran; 2 Department of Arid and Mountainous Regions Reclamation, Faculty of Natural Resources, University of Tehran, Tehran, Iran; 3 Future Technology Research Center, National Yunlin University of Science and Technology, 123 University Road, Section 3, Douliou, 64002 Yunlin Taiwan; 4 Computer Science Department, Boise State University, 777 W Main St, Boise, ID 83702 USA; 5 Department of Civil Engineering, Islamic Azad University, Central Tehran Branch, Tehran, P.O. Box 13185, Iran; 6 John von Neumann Faculty of Informatics, Obuda University, 1034 Budapest, Hungary

**Keywords:** Environmental sciences, Natural hazards, Chemistry

## Abstract

In the present study, the simultaneous removal of malachite green (MG) and auramine-O (AO) dyes from the aqueous solution by NaX nanozeolites in a batch system is investigated. Taguchi method and response surface methodology (RSM) were used to optimize and model dye removal conditions. In order to do so, the effect of various factors (dyes concentration, sonication time, ionic strength, adsorbent dosage, temperature, and pH of the solution) on the amount of dye removal was evaluated by the Taguchi method. Then, the most important factors were chosen and modeled by the RSM method so as to reach the highest percentage of dye removal. The proposed quadratic models to remove both dyes were in good accordance with the actual experimental data. The maximum removal efficiencies of MG and AO dyes in optimal operating conditions were 99.07% and 99.61%, respectively. Also, the coefficients of determination (R^2^) for test data were 0.9983 and 0.9988 for MG and AO dyes, respectively. The reusability of NaX nanozeolites was evaluated during the adsorption process of MG and AO. The results showed that the adsorption efficiency decreases very little up to five cycles. Moreover, NaX nanozeolites were also applied as adsorbents to remove MG and AO from environmental water samples, and more than 98.1% of both dyes were removed from the solution in optimal conditions.

## Introduction

Pollution of water resources has become an economic problem since industrial factories have been increasing and water resources are limited^1,2^. Numerous industries, such as textiles, pharmaceuticals, and papermaking, produce large volumes of dye effluents. It is reported that about 10,000 types of commercial dyes with a volume of more than 700,000 tons are produced worldwide. In fact, about 20% of these dyes enter the aqueous medium due to the lack of proper stabilization of dye molecules on the fibers and the inefficiency of dyeing factories in wastewater treatment. Studies have shown that most of these dyes are toxic, allergenic, carcinogenic, and mutagenic to humans and various organisms^3,4^.

Malachite green (MG) is a toxic cationic dye originally used in the dyeing industry for materials such as silk, leather, and paper. This dye was first used in the fisheries industry in 1933^5^. Because it was too inexpensive and effective in eradicating aquatic infections. This dye was used too much in many countries. On the other hand, as a result of using this dye, many destructive effects such as carcinogenicity and mutagenicity have been reported in various organisms, especially mammals^6–8^.

Auramine-O (AO) is a water-soluble cationic dye. AO dye is one of the dyes used in the textile, carpet, and leather industries. Studies have shown that this dye is converted to biotransformation in human organs and increases the risk of bladder cancer^9,10^. Accordingly, efforts should be made to improve methods that reduce or eliminate these dyes from the aqueous environment.

There are different ways to remove the dye, such as reverse osmosis, ultra-filtration, ion exchange, and adsorption^11–17^. Adsorption is one of the acceptable techniques to reduce the concentration of dissolved dyes in aqueous solutions. The advantages of this technique include simplicity in operation, cheapness, and flexibility compared to other separation techniques^18,19^.

Various adsorbents such as eggshell^20^, activated carbon^21^, bentonite^22^, shrimp shell^23^, and zeolite^24^ have been used to remove dye effluents. Recently, researchers have applied inexpensive adsorbents with high adsorption potential with high adsorption power and do not harm the environment. Thus, economic problems and the recovery of adsorbents have made researchers focus on inexpensive adsorbents such as zeolites.

Zeolites are generally divided into natural and synthetic. Natural zeolites are mostly found in volcanic rocks, but they are not produced a lot because they are not economical to extract. Synthetic zeolites are far better than their natural counterparts due to a special type, high purity, easy commercial-scale access, and fixed and controllable pore sizes. The most popular synthetic zeolites include A, X, Y, and ZSM-5^25,26^. In synthetic zeolites, there are fundamental changes in the properties of these materials as the particle size is reduced from micrometers to nanometers, which is very effective on the function of zeolites in catalytic applications and separation. As the particle size decreases, the ratio of the number of outer atoms to the material increases rapidly, resulting in an increase in the outer surface area and significant surface activity^27,28^. Nowadays, the use of zeolites as adsorbents in the adsorption process has received much attention.

Arabkhani et al. (2021) have recently used magnetic GO/ZIF-8/γ-AlOOH-NC as a novel and effective adsorbent to remove diclofenac from hospital effluents. Optimal conditions of diclofenac removal were evaluated by the RSM method. The reusability results revealed that the reuse of the adsorbent up to 5 times caused no significant reduction in its adsorption capacity. They also investigated the magnetic efficiency of GO/ZIF-8/γ-AlOOH-NC in diclofenac removal from simulated hospital effluents containing various drugs and organic and inorganic substances. They reported that the magnetic GO/ZIF-8/γ-AlOOH-NC could be promising as an efficient adsorbent for diclofenac removal from wastewater^29^.

A research was conducted on the application of ZSM-5 zeolite to remove malachite green from aqueous solutions. Optimum condition zeolite dosage 5 wt%, pH  10, initial dye concentration of 10 mg L^−1^, and temperature 25 °C was obtained. Also, 99.12% dye removal was achieved in optimal conditions^30^.

In another study, nano ZSM-5 zeolite (nZSM-5) synthesized from rice husk ash to remove crystal violet from aqueous solutions was carried out by Sivalingam and Sen (2020) effectiveness of nZSM-5 in removing crystal violet from aqueous solutions was investigated. Maximum dye removal (99.99%) in conditions adsorbent dosage of 100 mg, pH of 8, initial CV dye concentration of 100 mg L^−1^, and sonication time of 30 min were obtained^31^.

Sivalingam and Sen used nanozeolite X to remove ions such as Cu^2+^, Zn^2+^, Pb^2+^, Cd^2+^, Ca^2+^, Ni^2+^, Mg^2+^, and various dyes such as crystal violet methylene blue, Congo red, and indigo carmine. The results showed that the maximum adsorption capacity of metals was obtained for Pb^2+^ 196.24 mg g^−1^ and methylene blue 193.45 mg g^−1^. Therefore, nanozeolite X can be used as a highly efficient adsorbent to remove various dyes and ions^32^.

The ultrasonic process has been considered as an efficient and advanced technology in various fields of science to help eliminate contaminants in water^33^. Theory to explain how ultrasound breaks chemical bonds involves the formation, growth, and eventual destruction of a bubble that forms within a liquid. This phenomenon, known as acoustic cavitation, creates an environment with a pressure of up to 10 pascals. The phenomenon of acoustic cavitation increases mass transfer, increases the adsorption permeability into the adsorbent, increases process efficiency, and reduces time^34,35^.

Generally, experiments and factors affecting the process are carried out in the form of one-factor-at-a-time. Different factors in this method are done by changing the effective factor studied in a range of levels and keeping other factors constant. To achieve optimal conditions by this method, the tests must be repeated for all factors, which leads to a substantial increase in the number of tests. In order to cope with the limitations, various experimental design methods based on mathematical and statistical techniques have been developed, including the Taguchi method and response surface methodology (RSM)^36,37^. Taguchi method includes experimental design method to determine the effect of factors on the response and to obtain the optimal process conditions. One of the main advantages of this method is providing optimal conditions with the minimum number of experiments using orthogonal arrays, leading to cost reduction^38,39^. RSM is very useful to design experiments and analyze data to lead to a purposeful and reliable conclusion. RSM is a particular set of mathematical and statistical methods used to design experiments, construct models, evaluate optimal conditions, effect independent variables on dependent variables, and obtain optimal conditions for multiple responses simultaneously. Also, the graphs presented in the RSM method are three-dimensional, which allows showing the change of all factors in one graph^40,41^. This study aimed to remove toxic dyes by NaX nanozeolites as well as. The variables were first examined based on the Taguchi method, and the most important ones were selected. In the next step, these variables were modeled using the RSM method to determine the optimal conditions.

## Experimental section

### Materials and instruments

**Figure 1:**
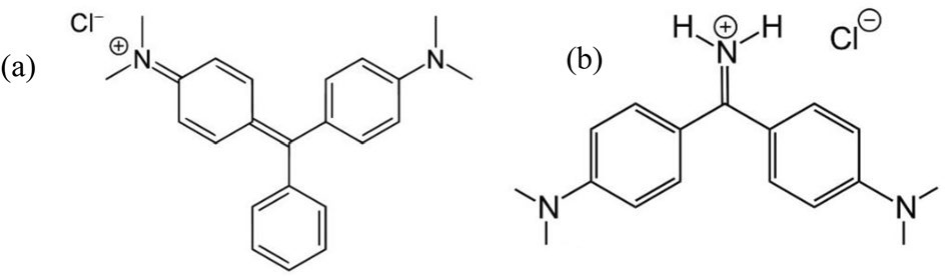
The chemical structure of **(a)** MG, and **(b)** AO.

All materials used, such as sodium aluminate, sodium hydroxide, tetraethylorthosilicate, hydrochloric acid, and other chemicals, were used without further refining and were supplied from Aldrich or Merck companies. The stock solution of the dyes was prepared by dissolving the solid substance of each dye in distilled water. Other solutions in this study were prepared by diluting the stock solution and used immediately in adsorption experiments. UV/Vis spectrophotometer (2120 UV plus, Optizen) was used to detect dyes at maximum wavelength of malachite green (MG) (λ_max_ = 620 nm) and auramine-O (AO) (λ_max_ = 430 nm). Sodium hydroxide (1 M) and hydrochloric acid (1 M) solutions were used to adjust the pH. The chemical structures and descriptions of the dyes are shown in Fig. 1 and Table 1, respectively.

**Table 1:** The characteristics of the dyes.

Dye	Dye formula	λ_max_ (nm)	Molecular weight (g mol^−1^)
Malachite green	C_23_H_25_N_2_	620	364.911
Auramine-O	C_17_H_22_ClN_3_	430	303.83

### Preparation of NaX nanozeolites

In order to synthesize NaX nanozeolites, molecular composition (5.5 Na_2_O: 1.0 Al_2_O_3_: 4.0 SiO_2_: 190 H_2_O) was used. Also, 7.22 g of sodium hydroxide was calculated according to the molar composition and dissolved in a plastic bottle (250 ml) with distilled water. Then, the solution was divided into two equal parts, and in the first part, 0.16 g of sodium aluminate and the second part 5.22 g of Tetraethyl orthosilicate were added. The two solutions, including aluminate and silicate solutions, were then mixed and placed in an ice-water bath. Hydrothermal crystallization was done by shaking with a shaker for 3 days. Furthermore, the obtained powder was recovered by centrifugation for 5 min at 3500 rpm. The synthesized NaX nanozeolites were washed several times with distilled water to bring the pH below 8 and finally dried at room temperature for 1 day^42,43^. The synthesized NaX nanozeolites have been characterized by X-ray diffraction (XRD) using Cu K_a_ as the radiation source, scanning electron microscopy (SEM), and Brunauer Emmett Teller (BET) adsorption/desorption isotherms for surface area analysis.

### Experimental design

#### Taguchi method

Taguchi is a method that reduces the number of experiments by minimizing the interference of uncontrolled factors, which is used as a mathematical technique. By creating orthogonal arrays and matching many factors, Taguchi identifies minor variables in the shortest amount of time. The orthogonal arrays are shown in Table 2. Factors studied include temperature (25–35 °C), solution pH (3–9), adsorbent (100–300 mg), ionic strength (0–6 w/v%), dye concentration (6–10 mg L^-1^) and sonication time (3–9 min). Applying the Taguchi method, only 27 experiments are required to obtain the optimal levels of the variables (Table 2), while the most accurate optimization method for a complete study of seven variables at three levels requires 2187 experiments (3^7^ = 2187), which is practically time-consuming. Therefore, the Taguchi method can reduce the number of tests, reduce time, decrease costs, and determine important factors in a short time. Taguchi uses the signal-to-noise ratio in measurable amounts of qualitative characteristics according to the purpose of the experiments. The signal-to-noise ratio (S/N) is obtained by Eq. (1):

**Table 2:** Process variables with their values.

Variables	Unit	Level 1	Level 2	Level 3
Adsorbent dosage	mg	100	200	300
MG concentration	mg L^−1^	6	8	10
AO concentration	mg L^−1^	6	8	10
Ionic strength	(w/v%)	0	3	6
pH of the solution	–	4	6	8
Temperature	°C	25	27	30
Sonication time	min	3	6	9

1 $$\frac{S}{N}=-10 \mathrm{log}(\frac{1}{n}\sum_{k=1}^{n}\frac{1}{{y}_{i}^{2}})$$

In this equation, n is the number of experiments, and y is the response of the variables^44^.

#### Response surface methodology (RSM)

RSM is a set of mathematical and statistical methods determining the relationship between one or more responses to several variables. In chemistry, many phenomena are modeled based on their theories. However, many phenomena do not have a satisfactory mathematical model due to their dependence on many controlling factors, unknown mechanisms, and mathematical complexity. In such cases, the use of experimental modeling methods such as the response level method is effective. In the central composite design (CCD)-based RSM, variables are examined at five levels. Low levels (-α) and high levels (+ α) are entered into the software by the operator, and the software provides other levels. According to the results obtained in the Taguchi method in “Taguchi method”. Taguchi method, in this step, five factors that were of great importance were examined. These factors were the amount of adsorbent, dye concentration, sonication time, and pH solution (Table 3). The percentage of dye removal was considered as the response variable. The equation that can be used in the response surface method is the polynomial quadratic equation. The responses must conform to Eq. (2) in order to use it.

2 $${\text{Y}} = \beta_{0} + \mathop \sum \limits_{i = 1}^{k} \beta_{i} X_{i} + \mathop \sum \limits_{i = 1}^{k} \beta_{ii} X_{i}^{2} + \mathop \sum \limits_{i \le j}^{k} \mathop \sum \limits_{j}^{k} \beta ijX_{i} X_{j} + e$$

Where k is the number of variables, *β*_*0*_ is the model constant, *β*_*i*_ are the coefficients of linear factors, *β*_*ij*_ and *β*_*ii*_ are the coefficients of the factors that interact with each other, ɛ the remaining values are related to random error, *X*_*i*_ and *X*_*j*_ are the variables^45^.

**Table 3:** Levels of the variable in the RSM.

Variables	Symbol	Unit	Levels				
			− α	Lower	Central	Upper	+ α
Adsorbent dosage	A	mg	200	250	300	350	400
pH of the solution	B	–	4	6	8	10	12
MG concentration	C	mg L^−1^	2	4	6	8	10
AO concentration	D	mg L^−1^	2	4	6	8	10
Sonication time	E	Min	3	6	9	12	15

### Analytical methods

In order to study the efficiency of NaX nanozeolites to remove MG and AO dyes, batch experiments were performed. The experiments were designed by the CCD method. For this reason, at room temperature, in a centrifuge tube, 25 mL of a solution containing both dyes (4 mg L^−1^) was added. Then, 347 mg of NaX nanozeolites were added to the sample solution. The pH of the solution was adjusted to 8. The solution was placed in an ultrasonic bath for 11.5 min and centrifuged at 3000 rpm for 5 min. Finally, the supernatant was removed and transferred to UV/Vis cells to determine the amount of residual concentration and to calculate the percentage of dye removal of MG and AO, and the adsorption of solutions for MG and AO was read at 620 nm and 430 nm, respectively. In these experiments, Eq. (3) was used to determine the percentage of dye removal.

3 $$\% {\text{ Removal}}\, = \left( {\frac{{C_{0} - C}}{{C_{0} }}} \right) \times 100$$

C_0_ and C are the initial and final concentrations of the desired dye in terms of mg L^−1^, respectively^46^.

## Results and discussion

### Characterization of the NaX nanozeolites

**Figure 2:**
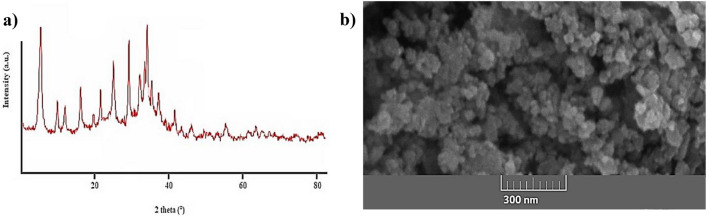
**(a)** XRD pattern of nanozeolite-X, **(b)** SEM image of the corresponding sample.

Figure 2a shows the XRD pattern of the sample, and XRD analysis shows that high purity NaX zeolite phase without phase interference has been synthesized in the above phase method (JCPDS no. 39-0218)^47,48^. The average size of the crystals synthesized using the Scherer equation was in the range of 40–70 nm. The crystal size indicates that the synthesis of NaX zeolite in nanometer dimensions has been successful. Morphological analysis of NaX nanozeolites was performed using SEM. The SEM image of the synthesized zeolite sample (Fig. 2b) shows that the particle sizes are in the range between 50 and 150 nm. Also, the adsorption and desorption porosity was measured by the BET analysis. The BET surface area, calculated average particle size, and total pore volume of synthesized NaX nanozeolites were found as 852.5 m^2^/g, 69.42 nm, 0.304 cm^3^/g, respectively.

### Determination of pH_PZC_

pH_PZC_ is the point at which the adsorbent surface charge is neutral. Thus, at a pH above this point, the adsorbent surface has a negative charge, and at a lower pH, the surface charges become positive. In order to determine pH_PZC_, 10 mL of NaCl solution (0.1 M) was poured into separate test tubes, and the solutions were adjusted to different pH (2–12). Hydrochloric acid (1 M) and sodium hydroxide (1 M) were used to adjust the pH. Then, 0.3 g of adsorbent was added to the solutions, and the samples were placed in a shaker at 150 rpm. After 24 h, the adsorbents were separated from the solution, and the solutions' pH was measured again. The pH_PZC_ was found to be 6.5 (Fig. S1).

### Response factors based on the Taguchi

The experimental design was performed by the Taguchi method in Minitab software version 19. In order to estimate the most important factors in the removal of MG and AO dyes by NaX nanozeolites by the Taguchi method, 7 factors were investigated at 3 levels. These levels were initially obtained by trial and error. The software proposed the orthogonal table L27 (7 factors in 3 levels) to design the above experiment, which includes 27 experiments. Taguchi test design table for removing MG and AO dyes is given in Table S1.

**Figure 3:**
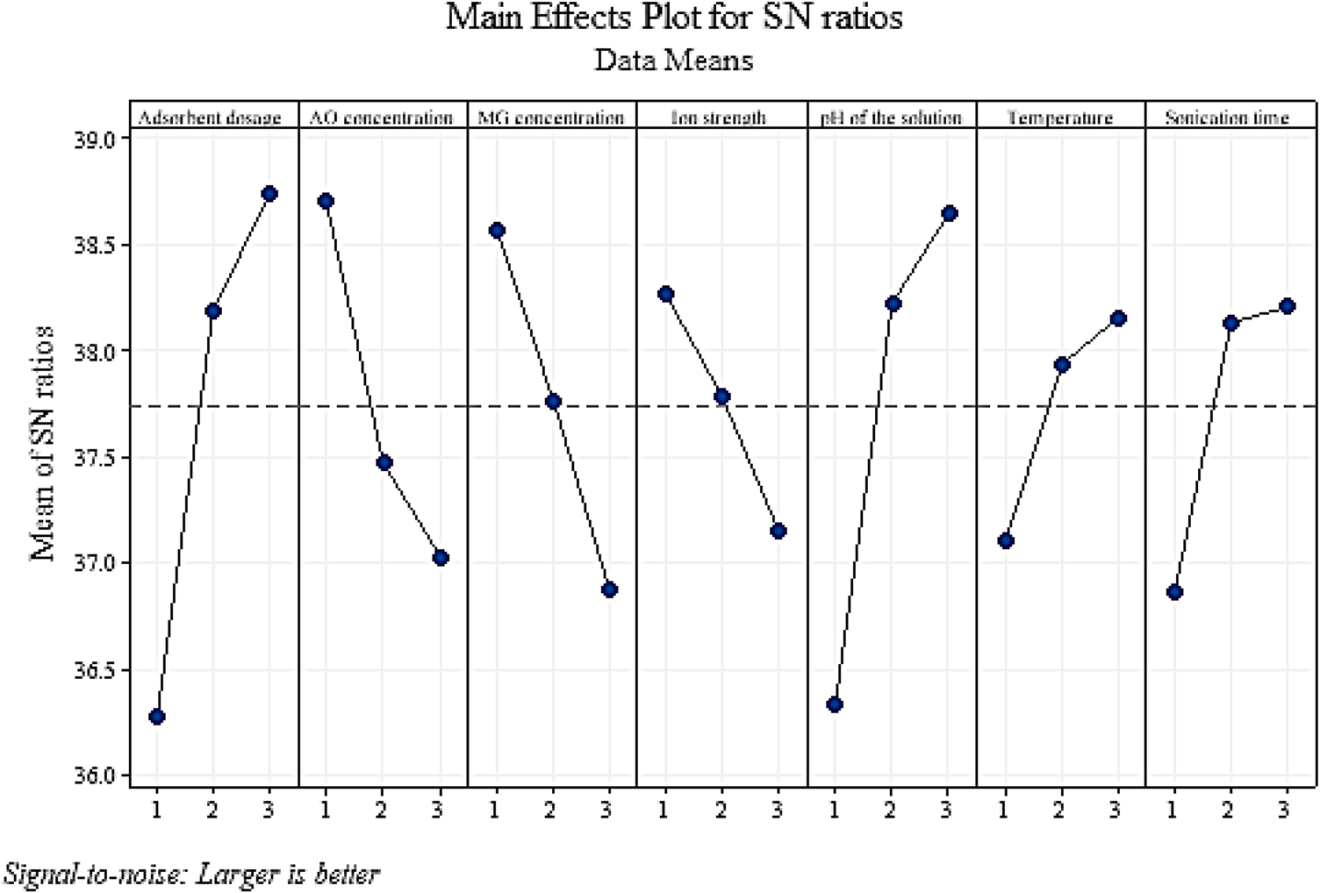
Signal to noise ratios for removal MG and AO.

The change in each of the factors indicates the importance of the factor in the process. In Table 4, the effect of each factor at each level was calculated independently by the software, and finally, according to the differences created in each factor, the importance of each was investigated. In MG and AO dye removal experiments, the amount of adsorbent has the first effect on the adsorption process, pH comes as the second factor, MG dye concentration the third, AO dye concentration the fourth, sonication time the, ionic strength the sixth, and finally the temperature is the seventh factor. Based on the results of Table 4, the variables of adsorbent amount, solution pH, dye concentration, and sonication time were selected as effective variables for optimization and modeling by the RSM method. Therefore, the variables of temperature and ionic strength were omitted. Because as shown in Fig. 3 and Table 4, the temperature has little effect on the process compared to other factors and was maintained at 25 °C in subsequent optimal experiments. Increasing ionic strength also reduces the adsorption of dyes by NaX nanozeolites. This can be attributed to preventing dye molecules from approaching the active sites of adsorption^49^. The same effect has been reported in the literature for some cationic dyes, such as the adsorption of methylene blue by sludge ash, methylene blue, and crystal violet by palm kernel fiber^50,51^.

**Table 4:** Response table for signal to noise ratios for MG and AO.

Level	Adsorbent dosage (mg)	AO concentration (mg L^−1^)	MG concentration (mg L^−1^)	Ionic strength (w/v%)	pH of the solution	Temperature (°C)	Sonication time (min)
1	36.26	38.69	38.55	38.25	36.33	37.11	36.87
2	38.19	37.47	37.77	37.79	38.23	37.94	38.13
3	38.74	37.03	36.87	37.16	38.63	38.14	38.19
Delta	2.48	1.65	1.67	1.09	2.30	1.03	1.32
Rank	1	4	3	6	2	7	5

### Significant variable optimization by RSM

In this section, Design Expert statistical software version 10 was used to execute the CCD design and analyze the resulting data. Software output includes main effects, their interactions, quadratic equation, and statistical graphs. To perform experiments by the CCD method, the software designed 32 experiments. Table 5 shows the order of these 32 experiments. Method of analysis in “Analytical methods”. Analytical methods are provided. The answer to each row of the experiment is also given.

**Table 5:** CCD design of variables and their corresponding experimental and predicted removal of MG and AO.

Run No	Variables					Responses			
						%R_MG_		%R_AO_	
	A	B	C	D	E	Actual	Predicted	Actual	Predicted
1	350	6	8	4	12	63.56	63.75	73.44	73.28
2	350	10	8	4	6	92.97	93.56	87.73	87.81
3	300	8	2	6	9	84.50	83.88	90.62	90.89
4	300	8	6	2	9	77.76	76.28	87.08	87.68
5	300	4	6	6	9	29.18	29.49	38.37	38.97
6	300	8	6	6	9	82.15	82.00	92.25	92.43
7	250	6	4	4	12	57.62	57.92	61.59	61.28
8	400	8	6	6	9	87.39	86.99	87.85	87.81
9	300	12	6	6	9	62.91	61.99	79.23	78.35
10	350	10	8	8	12	57.86	57.77	96.40	96.98
11	250	6	8	4	6	34.29	34.53	43.91	43.67
12	300	8	6	10	9	43.47	44.35	62.48	61.61
13	300	8	6	6	15	84.06	83.95	89.73	89.33
14	300	8	6	6	9	84.44	82.00	94.26	92.43
15	350	10	4	4	12	98.68	99.33	99.03	99.04
16	300	8	10	6	9	56.85	56.86	74.86	74.31
17	200	8	6	6	9	54.17	53.96	48.06	47.82
18	250	10	8	8	6	37.94	37.90	48.54	49.04
19	300	8	6	6	9	80.94	82.00	90.69	92.43
20	300	8	6	6	9	79.75	82.00	91.37	92.43
21	350	6	4	8	12	85.39	85.15		62.29
22	350	6	4	4	6	52.46	52.90		84.43
23	300	8	6	6	9	82.73	82.00		92.43
24	350	6	8	8	6	27.69	27.39		43.91
25	250	6	8	8	12	46.09	45.65		46.25
26	300	8	6	6	9	81.38	82.00		92.43
27	350	10	4	8	6	58.07	58.23		78.71
28	250	10	4	4	6	75.73	76.43		76.74
29	300	8	6	6	3	58.76	58.27		74.55
30	250	10	8	4	12	56.98	57.43		72.34
31	250	10	4	8	12	56.35	56.37		64.56
32	250	6	4	8	6	39.90	39.71		52.59

A: adsorbent dosage (mg), B: pH of the solution, C: MG concentration (mg L^−1^), D: AO concentration (mg L^−1^) and E: sonication time (min).

A reliable method for evaluating the quality of a matched model is the analysis of variance (ANOVA). In ANOVA, the share of variance of each factor is compared with the variance caused by random errors in measurement. In fact, the significance of regression can be examined through this comparison. Significance of regression is performed by comparing the regression variance to the variance of the residuals with the Fisher distribution (F-test). If this ratio is greater than the critical value of F, the mathematical model is consistent with the experimental data. If the calculated p-value for each of the factors is less than 0.05, it indicates the effectiveness of that factor, and if it is more than 0.05, it means that the change of that factor does not affect the values. The parameters for MG and AO dyes are given in Table 6. The correlation coefficients (R^2^) were 0.9983 and 0.9988, and adj-R^2^ were 0.9953 and 0.9967 for MG and AO, respectively. High values of R^2^ and adj-R^2^ confirm the model's ability to make a convincing estimate of the response.

**Table 6:** Analysis of variance for the quadratic polynomial model for removal of MG and AO.

Source	DF	MG				AO			
		Sum of squares	Mean square	F-value	P-value	Sum of squares	Mean square	F-value	P-value
Model	20	12,040.00	602.00	329.35	< 0.0001	10,570.00	528.50	473.63	< 0.0001
A	1	1637.13	1637.13	895.65	< 0.0001	2399.00	2399.00	2149.93	< 0.0001
B	1	1585.03	1585.03	867.14	< 0.0001	2325.98	2325.98	2084.49	< 0.0001
C	1	1095.12	1095.12	599.12	< 0.0001	412.43	412.43	369.61	< 0.0001
D	1	1529.29	1529.29	836.65	< 0.0001	1019.34	1019.34	913.51	< 0.0001
E	1	989.19	989.19	541.17	< 0.0001	327.60	327.60	293.59	< 0.0001
AB	1	53.95	53.95	29.51	0.0002	98.75	98.75	88.50	< 0.0001
AC	1	0.20	0.20	0.11	0.7482	28.60	28.60	25.63	0.0004
AD	1	73.62	73.62	40.27	< 0.0001	27.80	27.80	24.91	0.0004
AE	1	127.24	127.24	69.61	< 0.0001	12.87	12.87	11.53	0.0060
BC	1	26.68	26.68	14.59	0.0028	103.07	103.07	92.37	< 0.0001
BD	1	692.74	692.74	378.99	< 0.0001	7.52	7.52	6.74	0.0249
BE	1	542.42	542.42	296.75	< 0.0001	30.61	30.61	27.43	0.0003
CD	1	69.89	69.89	38.24	< 0.0001	31.39	31.39	28.13	0.0003
CE	1	101.40	101.40	55.48	< 0.0001	303.89	303.89	272.34	< 0.0001
DE	1	230.28	230.28	125.98	< 0.0001	66.14	66.14	59.27	< 0.0001
A^2^	1	243.48	243.48	133.20	< 0.0001	1110.81	1110.81	995.48	< 0.0001
B^2^	1	2410.32	2410.32	1318.65	< 0.0001	2090.76	2090.76	1873.69	< 0.0001
C^2^	1	247.93	247.93	135.64	< 0.0001	177.15	177.15	158.76	< 0.0001
D^2^	1	862.43	862.43	471.82	< 0.0001	580.22	580.22	519.98	< 0.0001
E^2^	1	217.58	217.58	119.04	< 0.0001	201.74	201.74	180.79	< 0.0001
Residual	11	20.11	1.83			12.27	1.12		
lack of fit	6	7.09	1.18	0.45	0.8180	4.29	0.71	0.45	0.8222
Pure error	5	13.02	2.60			7.99	1.60		
Cor total	31	12,060.11				10,582.28			

Model summary statistics									
MG				AO					
R^2^	Adj-R^2^	Pred-R^2^	Adequate precision	R^2^	Adj-R^2^	Pred-R^2^	Adequate precision		
0.9983	0.9953	0.9832	65.69	0.9988	0.9967	0.9882	70.19		

A: adsorbent dosage (mg), B: pH of the solution, C: MG concentration (mg L^−1^), D: AO concentration (mg L^−1^) and E: sonication time (min).

As can be seen in Table 6, the value of p for linear and interaction factors is less than 0.05. From the value of p related to nonconformity, it can be deduced that the equation obtained is consistent with the experimental data. The mathematical model was presented as a second-order polynomial relation in coded form (A, B, etc.) to describe each deletion efficiency response (%R) for each of the dyes in Eqs. (4) and (5).

4 $${\text{R}}_{{{\text{MG}}}} \, = \,\, + \,{82}.00\, + \,{8}.{26}*{\text{A}}\, + \,{8}.{13}*{\text{B }} - {6}.{75}*{\text{C }} - {7}.{98}*{\text{D}}\, + \,{6}.{42}*{\text{E}}\, + \,{1}.{84}*{\text{AB}}\, + \,0.{11}*{\text{AC }} - {2}.{14}*{\text{AD}}\, + \,{2}.{82}*{\text{AE}}\, + \,{1}.{29}*{\text{BC }} - {6}.{58}*{\text{BD }} - {5}.{82}*{\text{BE }} - {2}.0{9}*{\text{CD }} - {2}.{52}*{\text{CE}}\, + \,{3}.{79}*{\text{DE }} - {2}.{88}*{\text{A}}^{{2}} - {9}.0{6}*{\text{B}}^{{2}} - {2}.{91}*{\text{C}}^{{2}} - {5}.{42}*{\text{D}}^{{2}} - {2}.{72}*{\text{E}}^{{2}}$$

5 $$\chi {\text{R}}_{{{\text{AO}}}} \, = \,\, + \,{92}.{43}\, + \,{1}0.00*{\text{A}}\, + \,{9}.{84}*{\text{B }} - {4}.{15}*{\text{C }} - {6}.{52}*{\text{D}}\, + \,{3}.{69}*{\text{E}}\, + \,{2}.{48}*{\text{AB}}\, + \,{1}.{34}*{\text{AC }} - {1}.{32}*{\text{AD}}\, + \,0.{9}0*{\text{AE}}\, + \,{2}.{54}*{\text{BC}}\, + \,0.{69}*{\text{BD}}\, + \,{1}.{38}*{\text{BE}}\, + \,{1}.{4}0*{\text{CD}}\, + \,{4}.{36}*{\text{CE}}\, + \,{2}.0{3}*{\text{DE }} - {6}.{15}*{\text{A}}^{{2}} - {8}.{44}*{\text{B}}^{{2}} - {2}.{46}*{\text{C}}^{{2}} - {4}.{45}*{\text{D}}^{{2}} - {2}.{62}*{\text{E}}^{{2}}$$

In Eqs. (4) and (5), the parameters of adsorbent amount (A), pH of solution (B), MG concentration (C), AO concentration (D), and sonication time (E).

**Figure 4:**
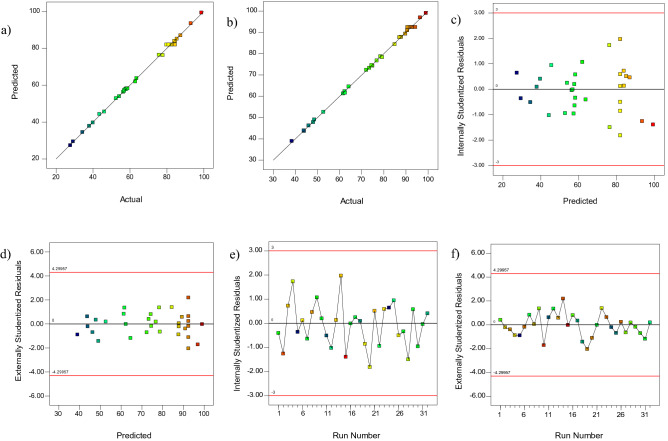
Plot of predicted value versus actual value **(a)** MG and **(b)** AO, plots of residuals for removal of **(c,e)** MG and **(d,f)** AO.

**Figure 5:**
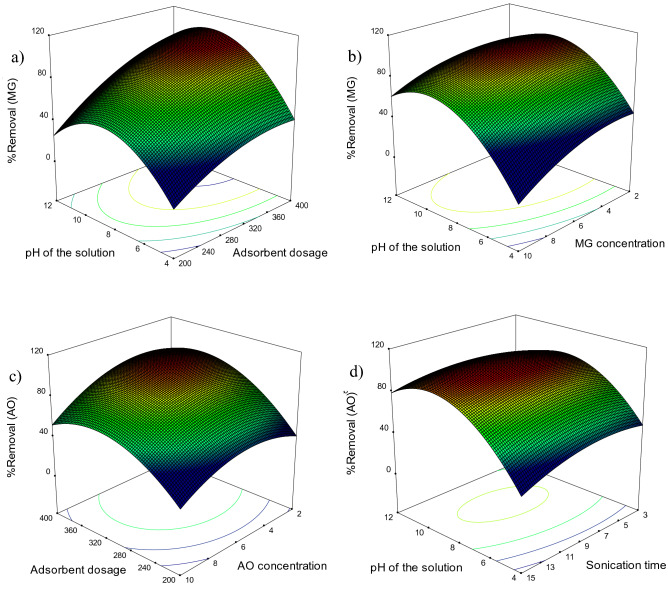
Response surface plots of removal of **(a,b)** MG and **(c,d)** AO.

Residual diagrams help to interpret the results accurately. Assuming that the errors are normally distributed and independent of each other, residual probability diagrams (Fig. 4a,b) are an essential diagnostic tool to identify and explain systematic deviations. Also, the residual probability graph also shows that the error variance is homogeneous^28^. In this diagram, the closer the points are to the line, the less error there is. As shown in Fig. 4a,b are points close to the line. In Fig. 4c–f, the better the distribution of points at the top and bottom of the axis is the same (i.e., the probability of positive and negative error is the same, and the test error is not a systematic error). As shown in Fig. 4c–f, there is no systematic error. Figure 5 shows the three-dimensional diagrams of the interaction effect. Three-dimensional diagrams of surface response are a function of two independent parameters that keep all other parameters at constant levels. These diagrams can provide information about the relationship between the two parameters and are useful in understanding the main effects and interaction effects of the two parameters. Figure 5a shows the interaction of the two parameters of solution pH and the amount of adsorbent on MG dye removal. The MG dye removal goes up with increasing pH of the solution and increasing the amount of adsorbent. At pHs higher than pH_PZC_, which is 6.5 for NaX nanozeolites, the surface charge of the nanoparticles is negative. Thus, the adsorption of positively charged dye molecules due to electrostatic attraction increases. On the other hand, increasing the amount of adsorbent provides more adsorption sites for dye molecules to be adsorbed on the adsorbent surface. Therefore, the interaction of these two parameters, which causes the positive surface of nanoparticles and increases the adsorption sites, increases the adsorption. Similar results about an increase in the removal percentage with increasing pH (alkaline conditions or natural pH) and increasing the amount of adsorbent have also been reported for MG and AO dyes^33,52^. As shown in Fig. 5b,c, the amount of dye removal decreases with an increasing dye concentration of MG and AO. Decreasing the removal percentage at higher concentrations is due to the increase in dye concentration relative to the number of initial moles of dye available to the surface area. For a given amount of adsorbent, the total number of active sites available is constant, and as a result, the same amount of site absorbs the analyte, so as the initial dye concentration increases, the removal percentage decreases. Arabkhani and Asfaram (2020) used a novel three-dimensional magnetic polymer aerogel for the removal of MG dye and achieved similar results to this stage of this study, stating that the removal efficiency decreases with increasing the initial dye concentration^6^. Figure 5d shows the effect of sonication time on the amount of AO dye removal. As it is known, with increasing sonication time, the amount of dye removal should increase. This is because with increasing time, there is more opportunity for the dye and adsorbent molecules to be exposed. In a study entitled "Rapid removal of Auramine-O (AO) and Methylene blue (MB) dyes from aqueous solutions using ZnS:Cu nanoparticles as the adsorbent, Asfaram et al. (2015) found similar results to the present study and showed that the removal efficiency increased with increasing sonication time^10^.

### Optimization

Optimization in chemistry is used effectively and economically to reduce cost and time in multi-response methods. For this reason, following investigating the factors affecting the removal of MG and AO dyes by the Taguchi method, the conditions for removing the dye from the solution by RSM were optimized. According to the experiments performed in Taguchi design, the most important factors affecting the removal of MG and AO dyes in the method are solution pH, adsorbent mass, sonication time, the concentration of MG and AO dyes. These items were evaluated as the main factors (independent variables) in the RSM statistical design. The software presented the optimal values of each parameter and the relevant tests were performed. All stages of the experiment were carried out according to the Analytical methods section. Optimal values ​​and test results are shown in Table S2. It is observed that more than 99% of both dyes are removed from the solution by NaX nanozeolites in optimal conditions.

### Application to real samples

In order to study the efficiency of the method for the analysis of real samples, NaX nanozeolites were used as adsorbents to remove MG and AO from fish farms, tap water, and drinking water samples. For this reason, tests were performed in optimal conditions, in accordance with the method mentioned in “Analytical methods”. Environmental water samples were used instead of distilled water. After spectrophotometric determination of the remaining amount of dye, the percentage of simultaneous removal for MG and AO dyes was more than 98.1% in environmental water samples (Table S3). This means that NaX nanozeolites can remove significant amounts of MG and AO from environmental water samples.

### Interference studies

After obtaining the optimal conditions of effective parameters for removing the synchrony of MG and AO dyes, interference studies were carried out to evaluate the method's selectivity. In order to investigate the disturbance effect of different ions, different concentrations of disturbing ions were added to the solution and the steps were taken according to the method described in “Analytical methods”. Also, Analytical methods were applied (Removal conditions: 347 mg of NaX nanozeolites, pH: 8, the concentration of both dyes 4 mg L^−1^, centrifuge rate: 3500 rpm). The results are shown in Table S4. If the signal obtained in the presence of the disturbing ion differs by ± 5% from the signal in the absence of the disturbing ion, it indicates the degree of disturbance of the species on the decomposition signal. To determine the tolerance limit of the disturbing ion, a lower concentration of that species is examined to give an error value of ± 5%. According to the results, by adding almost high amounts of ions, no interference was observed on the decomposition signal. In this study and optimal conditions, the rate of dye removal in the presence of other ions was above 95%, which indicates the proper selectivity of NaX nanozeolites to both dyes despite the competitive effect of other ions.

### Desorption and reusability studies

**Figure 6:**
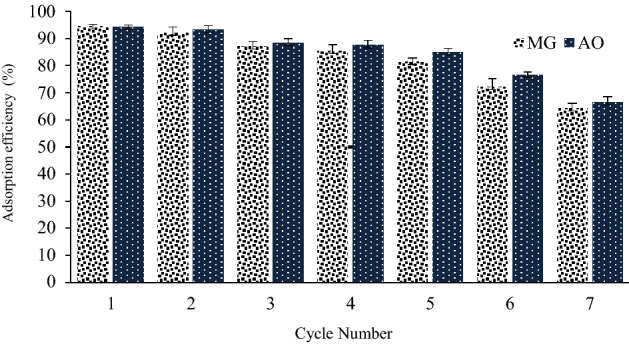
Effect of regeneration cycles on adsorption of dyes onto NaX nanozeolites.

The reuse of adsorbent could be considered as one of the important economic parameters. Therefore, the recyclability of NaX nanozeolites during the MG and AO adsorption process was evaluated. In this study, the NaX nanozeolites used were washed with 10 ml of methanol (0.01 M) and placed in an ultrasonic bath for 5 min. Finally, the amount of adsorption in each cycle was measured by spectrophotometry. The results in Fig. 6 show that up to 5 cycles, the adsorption efficiency decreases slightly. In general, this reduction can be due to adsorption degradation during adsorption–desorption cycles^53,54^.

### Comparison of adsorbents

The efficiency of the proposed method was evaluated with other methods for removing MG and AO dyes. The results are given in Table 7. This study showed that the developed method, compared to other methods, provides high removal of contamination (dye) in a short time from water samples. This method also has other advantages, such as the low number of tests, low operating costs, and optimization in the best possible way to achieve the highest efficiency (percentage of paint removal).

**Table 7:** Comparison of the NaX nanozeolites with other adsorbents for removal MG and AO.

Adsorbent	Dye	Adsorbent dosage	Concentration	pH	Time	Removal/adsorption capacity	Ref
Fe3O4/β-cyclodextrin-graphene oxide	MG	5 mg	100 mg L^−1^	7	2 h	98%	^55^
Amberlite XAD-4 (polymeric resin)	MG	1.5 g	10 mg L^−1^	10	10 min	93.12%	^56^
Sodium alginate-coated Fe3O4	MG	0.03 g	10	7	20 min	47.84 mg g^-1^	^57^
Polyacrylamide-g Chitosan γ-Fe2O3	MG	0.75 g	60 mg L^−1^	6	170 min	77%	^58^
Fe3O4/ activated carbon	MG	0.1 g	100 mg L^−1^	6	1 h	96%	^59^
NaX nanozeolites	MG	347 mg	4 mg L^−1^	8	11.5 min	99.07%	This work
Fe3O4/melamine-rich covalent organic polymer	AO	12 mg	10 mg L^−1^	6.5	4 min	107.11 mg g^-1^	^60^
Multiwall carbon nanotubes	AO	40 mg	10 mg L^−1^	7	1 h	97%	^61^
M5C^a^	AO	16 mg	5 mg L^−1^	9.5	8 min	17.95 mg g^-1^	^62^
Diospyros lotus seed powder	AO	0.1 g	20 mg L^−1^	6	2 h	26.95 mg g^-1^	^63^
Sugarcane bagasse	AO	0.05 g	200 mg L^−1^	8	9 h	682.8 mg g^-1^	^64^
Aca-NaAlg-cl-poly(AA)	AO	0.4 g	16 mg L^−1^	9	20 h	97.49%	^65^
Sodium dodecyl sulfate (SDS) functionalized magnetite nanoparticles	AO	20 mg	20 mg L^−1^	6.5	40 min	74%	^66^
NaX nanozeolites	AO	347 mg	4 mg L^−1^	8	11.5 min	99.61%	This work

^a^Metal organic framework-5 (MOF-5) and melamine-terephthaldehyde-based intergrade two imensional π-conjugated covalent organic framework (COF).

## Conclusion

The efficiency of NaX nanozeolites for simultaneous removal of malachite green (MG) and auramine-O (AO) dyes from aqueous solutions was investigated. The synthesized nanosorbents were characterized using SEM and XRD. The most important variables affecting the dye removal process were determined by the Taguchi method. These effective variables included solution pH, adsorbent mass, sonication time, MG, and AO dye concentrations and were optimized and modeled by CCD based on the RSM method. The optimal conditions obtained by RSM modeling included pH 8, ultrasound time of 11.5 min, an absorbent dose of 347 mg, and concentration of both dyes 4 mg L^−1^, and the highest dye removal (more than %99) was obtained for both dyes. Quadratic models for dye determination were statistically compared with values of R^2^˃ 0.99 and p < 0.0001, and the results showed that both models have reasonable accuracy. The results obtained for adsorption–desorption experiments showed that the adsorbent could be reused up to five times without a significant reduction in the percentage of dye removal. The methodmethod's efficiency for analyzing real samples containing MG and AO dyes also showed that the developed methodcould remove high amounts of dye contamination (%98.1) from complex samples.

## Supplementary Information

Supplementary Information.

## Data Availability

The authors declare that (the/all other) data supporting the findings of this study are available within the paper (and its supplementary information files).
