# Supplementary material for: Application of Taguchi method and response surface methodology into the removal of malachite green and auramine-O by NaX nanozeolites
Source: Sci Rep. 2021 Aug 6;11:16054. doi: 10.1038/s41598-021-95649-5 (PMC8346513; doi:10.1038/s41598-021-95649-5)
Supplement: Supplementary file 1 — Supplementary Information. [file 41598_2021_95649_MOESM1_ESM.docx]

**Supplementary Material**

**Fig. S1.** Point Zero charge of NaX nanozeolites.

**Table S1.** L_27_ orthogonal design and experimental results.

|  | Responses |  |  |  |  |  |  |  | Variables |  | Run No |
| --- | --- | --- | --- | --- | --- | --- | --- | --- | --- | --- | --- |
| % R_MG_ | % R_AO_ |  | Sonication time (min) | Temperature (°C) | pH of the solution | Ionic strength (w/v%) | MG concentration (mg L^-1^) | AO concentration (mg L^-1^) | Adsorbent dosage (mg) |  |  |
| 65.73 | 67.24 |  | 1 | 1 | 1 | 1 | 1 | 1 | 1 |  | 1 |
| 86.07 | 90.51 |  | 2 | 2 | 2 | 1 | 1 | 1 | 1 |  | 2 |
| 96.42 | 99.26 |  | 3 | 3 | 3 | 1 | 1 | 1 | 1 |  | 3 |
| 43.25 | 46.76 |  | 1 | 1 | 1 | 2 | 2 | 2 | 1 |  | 4 |
| 66.38 | 69.73 |  | 2 | 2 | 2 | 2 | 2 | 2 | 1 |  | 5 |
| 75.40 | 79.62 |  | 3 | 3 | 3 | 2 | 2 | 2 | 1 |  | 6 |
| 30.49 | 31.83 |  | 1 | 1 | 1 | 3 | 3 | 3 | 1 |  | 7 |
| 63.99 | 64.50 |  | 2 | 2 | 2 | 3 | 3 | 3 | 1 |  | 8 |
| 63.38 | 64.17 |  | 3 | 3 | 3 | 3 | 3 | 3 | 1 |  | 9 |
| 69.74 | 78.91 |  | 3 | 2 | 1 | 3 | 2 | 1 | 2 |  | 10 |
| 76.22 | 85.65 |  | 1 | 3 | 2 | 3 | 2 | 1 | 2 |  | 11 |
| 82.94 | 91.63 |  | 2 | 1 | 3 | 3 | 2 | 1 | 2 |  | 12 |
| 71.87 | 69.09 |  | 3 | 2 | 1 | 1 | 3 | 2 | 2 |  | 13 |
| 78.94 | 76.37 |  | 1 | 3 | 2 | 1 | 3 | 2 | 2 |  | 14 |
| 84.33 | 82.67 |  | 2 | 1 | 3 | 1 | 3 | 2 | 2 |  | 15 |
| 74.67 | 76.29 |  | 3 | 2 | 1 | 2 | 1 | 3 | 2 |  | 16 |
| 81.97 | 83.13 |  | 1 | 3 | 2 | 2 | 1 | 3 | 2 |  | 17 |
| 87.85 | 89.69 |  | 2 | 1 | 3 | 2 | 1 | 3 | 2 |  | 18 |
| 78.44 | 82.41 |  | 2 | 3 | 1 | 2 | 3 | 1 | 3 |  | 19 |
| 87.05 | 91.67 |  | 3 | 1 | 2 | 2 | 3 | 1 | 3 |  | 20 |
| 86.75 | 90.39 |  | 1 | 2 | 3 | 2 | 3 | 1 | 3 |  | 21 |
| 81.43 | 80.92 |  | 2 | 3 | 1 | 3 | 1 | 2 | 3 |  | 22 |
| 90.70 | 89.43 |  | 3 | 1 | 2 | 3 | 1 | 2 | 3 |  | 23 |
| 89.35 | 88.98 |  | 1 | 2 | 3 | 3 | 1 | 2 | 3 |  | 24 |
| 73.91 | 79.30 |  | 2 | 3 | 1 | 1 | 2 | 3 | 3 |  | 25 |
| 82.19 | 88.63 |  | 3 | 1 | 2 | 1 | 2 | 3 | 3 |  | 26 |
| 81.34 | 87.69 |  | 1 | 2 | 3 | 1 | 2 | 3 | 3 |  | 27 |

**Table S2.** Optimum conditions derived by CCD.

| Run | A (mg) | B | C (mg L^-1^) | D (mg L^-1^) | E (min) | % R_MG_ ± RSD | %R_AO_ ± RSD | % Predicted- R_MG_ | % Predicted- R_AO_ |
| --- | --- | --- | --- | --- | --- | --- | --- | --- | --- |
| 1 | 347 | 8 | 4 | 4 | 11.5 | 98.79 | 99.61 | 97.99 | 99.00 |
| 2 | 347 | 8 | 4 | 4 | 11.5 | 99.07 | 99.25 | 97.99 | 99.00 |
| 3 | 347 | 8 | 4 | 4 | 11.5 | 98.85 | 98.83 | 97.99 | 99.00 |

(A: adsorbent dosage, B: pH of the solution, C: MG concentration, D: AO concentration and E: sonication time)

**Table S3.** Average removal of MG and AO in the environmental water samples at optimum conditions (n=3).

| Real samples | R% ± RSD | |
| --- | --- | --- |
|  | MG | AO |
| Drinking water | 98.32 ±2.19 | 99.23 ±1.81 |
| Tap water | 99.86 ±3.09 | 98.11 ±1.67 |
| Fish farms | 98.48 ±3.57 | 98.73 ±2.50 |

(optimum conditions: water sample volume: 25 mL, MG concentration: 4 mg L^-1^, AO concentration: 4 mg L^-1^, sonication time: 11.5 min, pH: 8, adsorbent dosage: 347 mg).

**Table S4.** Tolerance limits of interfering ions in the removal of MG and AO.

| Tolerance limit (mg L^-1^) | Interference |
| --- | --- |
| 900 | $\mathrm{Na}^{+}$, $K^{+}$, $\mathrm{Br}^{-}$ |
| 600 | $\mathrm{NO}_{3}^{-}$, $\mathrm{ClO}_{4}^{-}$, $\mathrm{SO}_{4}^{2-}$ |
| 300 | $\mathrm{Mg}^{2+}$, $\mathrm{Cl}^{-}$, $\mathrm{Ca}^{2+}$ |
| 50 | $\mathrm{Cu}^{2+}$ |
